# *GBA* Variants Influence Motor and Non-Motor Features of Parkinson’s Disease

**DOI:** 10.1371/journal.pone.0167749

**Published:** 2016-12-28

**Authors:** Silvia Jesús, Ismael Huertas, Inmaculada Bernal-Bernal, Marta Bonilla-Toribio, María Teresa Cáceres-Redondo, Laura Vargas-González, Myriam Gómez-Llamas, Fátima Carrillo, Enrique Calderón, Manuel Carballo, Pilar Gómez-Garre, Pablo Mir

**Affiliations:** 1 Unidad de Trastornos del Movimiento, Servicio de Neurología y Neurofisiología Clínica, Instituto de Biomedicina de Sevilla, Hospital Universitario Virgen del Rocío/CSIC/Universidad de Sevilla, Seville, Spain; 2 Servicio de Medicina Interna, Instituto de Biomedicina de Sevilla, Hospital Universitario Virgen del Rocío/CSIC/Universidad de Sevilla, Seville, Spain; 3 Centro de Investigación Biomédica en Red de Epidemiología y Salud Pública (CIBERESP), Madrid, Spain; 4 Centro de Investigación Biomédica en Red sobre Enfermedades Neurodegenerativas (CIBERNED), Madrid, Spain; Oslo Universitetssykehus, NORWAY

## Abstract

The presence of mutations in glucocerebrosidase (*GBA*) gene is a known factor increasing the risk of developing Parkinson’s disease (PD). Mutations carriers have earlier disease onset and are more likely to develop neuropsychiatric symptoms than other sporadic PD cases. These symptoms have primarily been observed in Parkinson’s patients carrying the most common pathogenic mutations L444P and N370S. However, recent findings suggest that other variants across the gene may have a different impact on the phenotype as well as on the disease progression. We aimed to explore the influence of variants across *GBA* gene on the clinical features and treatment related complications in PD. In this study, we screened the *GBA* gene in a cohort of 532 well-characterised PD patients and 542 controls from southern Spain. The potential pathogeniticy of the identified variants was assessed using *in-silico* analysis and subsequently classified as benign or deleterious. As a result, we observed a higher frequency of *GBA* variants in PD patients (12.2% vs. 7.9% in controls, p = 0.021), earlier mean age at disease onset in *GBA* variant carriers (50.6 vs. 56.6 years; *p* = 0.013), as well as more prevalent motor and non-motor symptoms in patients carrying deleterious variants. In addition, we found that dopaminergic motor complications are influenced by both benign and deleterious variants. Our results highlight the fact that the impact on the phenotype highly depends on the potential pathogenicity of the carried variants. Therefore, the course of motor and non-motor symptoms as well as treatment-related motor complications could be influenced by *GBA* variants.

## 1. Introduction

Genetic susceptibility and toxic/environmental mechanisms are known factors which play a crucial role in the etiopathogenesis of Parkinson’s disease (PD). So far, several genes have been identified as causative of familial PD, such as *SNCA*, *LRRK2*, *PARK2*, *PINK1* and *DJ-1*. Furthermore, mutations and polymorphisms in other genes can increase the risk of suffering from PD, namely in susceptibility genes, including *GBA*, *MAPT*, *MC1R*, *ADH1C*, and HLA locus genes [1]. Former studies have demonstrated that *GBA* is at present one of the most prevalent genetic susceptibility factor for PD [2–7].

*GBA* is located in the 1q21-22 chromosome, and it encodes *γ-glucocerebrosidase*, a lysosomal enzyme that catalyzes the breakdown of the glycolipid glucosylceramide to ceramide and glucose. If this process fails, it could inhibit the correct lysosomal degradation of cellular α-synuclein, which may be a key mechanism in Lewy body formation [8]. Accordingly, *GBA* mutation carriers exhibit Lewy body pathology involvement in neocortical areas earlier than other sporadic PD cases without *GBA* variations [6].

Several studies have investigated the clinical features of *GBA*-related PD patients in recent years. Most of them concur that *GBA* mutation carriers have earlier disease onset, and are more likely to develop cognitive impairment or dementia and visual hallucinations [6,9–12], which may be related to pathological findings. Significant attention has already been paid to the phenotypic characterisation of PD patients with the most frequent pathogenic mutations in *GBA*, i.e. L444P and N370S [13]. However, there is evidence that other variants across the gene may influence the clinical profile and progression of the disease. Indeed, a recent study showed that PD patients with mutations or polymorphisms in *GBA* are at greater risk of progressing to Hoehn and Yahr Stage 3 [14]. Lastly, a recent large meta-analysis describes different risks for PD and age at onset for severe vs. mild *GBA* mutations [15]. Further research is therefore desirable, in order to clarify the role of *GBA* variants in clinical symptoms and disease progression based on its specific pathogenicity.

In this study we aimed to perform a complete sequence screening of *GBA* gene in our large cohort and studied the clinical features between variant carriers and non-carriers. More importantly, we sought to explore the course of the disease with regard to cardinal motor and non-motor clinical features such as dyskinesias, motor fluctuations, cognitive impairment and visual hallucinations as a function of the potential pathogenicity of the variants.

## 2. Methods

### 2.1. Patients and clinical data

In this study, we screened all coding exons and exon/intron boundaries of the *GBA* gene for mutations in 532 idiopathic PD patients and 542 unrelated healthy controls (HC) matched for age and sex. Patients were recruited from the Movement Disorder and General Neurology outpatient clinics at the Hospital Virgen del Rocío in Seville (Spain). PD was diagnosed using the United Kingdom Parkinson’s Disease Society Brain Bank criteria [16]. PD cases ranged from mild to severe. Patients within stages 1 up to 2.5 of Modified Hoehn and Yahr scale were considered as mild and severe those in 3–5 stages. The selection of controls was clinic-based, since the subjects were either spouses of the PD patients or patients from other outpatient clinics at the same hospital. All HC had no neurological disease. All subjects underwent a clinical interview at our centre and the clinical data was retrospectively obtained by consulting their previous medical records, including neurological and systemic symptoms for Gaucher disease (GD).

All subjects signed the informed consent and this study was approved by the local ehtic comite at the Hospital Universitario Virgen del Rocío.

We obtained an extensive set of clinical features for PD patients. These features included demographic and main symptom at onset data, motor symptoms, information about the pharmacologic treatment, and non-motor variables including neuropsychiatric and autonomic ones, and other non-motor symptoms. Based on the clinical notes, variables were collected as dichotomous (yes/no), quantitative or categorical.

The clinical evaluation was done between 2012–2015 and data concerning motor and non-motor symptoms and information about the pharmacologic treatment were included. The mean disease duration at evaluation was >10 years for the entire sample; this is important in this design since it indicates that none of the patients were retrospectively assessed over a longer period of time.

For some features for which the history of the patient at onset is of special interest, the date at symptom onset was retrospectively recorded so that we were able to perform a longitudinal assessment across groups. These included motor fluctuations, dyskinesias, cognitive impairment, visual hallucinations and depression.These data are protocolized collected in clinics. We defined the cognitive status following the Movement Disorders Task Force Criteria for dementia [17,18]. These criteria compile four axis to classify patients into possible or probable dementia. In this work, we considered within the cognitive impairment group those patients who fulfilled criteria for both, possible or probable dementia. To accomplish this purpose we gathered the information provided by patient and caregiver as well as the obtained results of the neuropsychological assessment. As screening instruments of cognitive impairment we performed either Mini Mental State Examination [19] or Parkinson’s disease Dementia Short Screen [20] tests. Concerning mood disorders, a careful history was done. The diagnosis was made in those situations with anxiety or depressive symptoms without relationship to treatment-related motor complications, as side effects to any drug or linked to any systemic condition. Regarding sleep disturbances, since a detailed sleep history may be sufficient in some populations for diagnosis of REM sleep behaviour disorders (RBD) [21], vivid dreams complains were targered as RBD.

### 2.2. Mutational screening and in silico analysis

#### 2.2.1 Mutational screening

Genomic DNA was isolated from the peripheral blood of each subject by standard or two automated methods (Maxwell 16 System, Promega Corporation, Madison, WI, USA; MagNA Pure LC, Roche Diagnostics, Indianapolis, IN), in compliance with established protocols. Polymerase chain reaction (PCR) primer couples were designed on the basis of the known genomic sequence (NG_009783.1). In order to avoid the amplification of the neighbouring pseudogene, *GBA* was first amplified in four large fragments that only and specifically amplified the functional gene but not the nearby pseudogene. For the mutational screening we study the isoform 1 of the *GBA* (NM_001005741. 2) which contains 12 exons, including a noncoding exon 1. The mutational screening of all exons and intron-exon boundaries was then performed using a combination of high-resolution melting (HRM) analysis and direct DNA resequencing. HRM reactions were performed on a LightCycler480 (LC480) instrument and HRM curve acquisition and analysis were performed using LC480 software version 1.3 (Roche Applied Science, Indianapolis, IN, USA). Samples showing abnormal melting profiles, including those with variants, were sequenced on both strands using the BigDye terminator cycle sequencing kit (Applied Biosystems, Foster City, CA, USA), analyzed using new PCR products and resolved on an ABI3500 genetic analyzer (Applied Biosystems).

We have adopted the conventional nomenclature, which refers to the processed protein and excludes the 39-residue signal peptide.

#### 2.2.2 *In-silico* analysis

The evolutionary conservation of the variants was examined using the PhyloP score [22] from the “PhyloP46wayAll” table (UCSC Table Browser; http://genome.usc.edu/cgi-bin/hgTables). The Grantham score, which quantifies changes to the chemical properties of the protein caused by amino acid substitutions, was also examined for each coding variant through the Grantham matrix table [23]. The pathogenic effect of the amino acid substitutions (AAS) on the three-dimensional protein structure and its impact on protein function was predicted using the servers: Polyphen-2 (http://genetics.bwh.harvard.edu.pph2/) [24], MutPred v.1.2 (http://mutpred.mutdb.org/) [25] and Mutation Taster (http://www.mutationtaster.org/) [26]. The effects of found intronic variants upon splicing signals were predicted using the following tools: Splice Site Prediction by Neural Network (NNSPLICE 0.9; (http://www.fruitfly.org/seq_tools/splice.html) [27] and Human Splicing Finder (HSF; http://www.umd.be/HSF/) [28]. Based on the information gathered, variants were classified as potentially deleterious and potentially benign. Those which reached a pathogenic predictive threshold in three or more bioinformatic tools were classified as deleterious non-synonymous variations. We defined synonymous variants as potentially deleterious if they were predicted to be pathogenic following the PhyloP score and Mutation Taster prediction. PD patients carrying GBA variants were therefore further subdivided into PD cases carrying potentially deleterious variants (Deleterious *GBA*-carriers) and PD cases carrying potentially benign variants (Benign *GBA*-carriers). Polyorphisms were identified accounting for dbSNP (http://www.ncbi.nlm.nih.gov/snp/) and those included in the public Human Gene Mutation Database (http://www.hgmd.org).

### 2.3. Statistical analysis

We performed a first analysis to analyze the differences in frequency of *GBA* variants between PD and HC groups in a case-control study. For this purpose we used a Chi-square test, and the odds ratio was calculated.

Within PD group we developed a case-series study. Demographic and clinical features were therefore compared pairwise using univariate analyses between the group of Deleterious *GBA*-carriers and the group of non-carriers, and between the group of Benign *GBA*-carriers and the group of non-carriers. A chi-square test was used to compare nominal variables (categorical and dichotomous) and a *t*-test to compare quantitative variables.

Cox regression was used to examine associations between GBA variant status and time-dependent outcomes such as motor fluctuations, dyskinesias, visual hallucinations and cognitive impairment. For this analysis we took disease duration at symptom onset for positive cases and disease duration at the review of the records for negative cases. Analyses were adjusted for potential confounding factors including age, sex and years with L-DOPA [29,30]. Years with L-DOPA were measured at symptom onset, i.e. fluctuations, dyskinesias, visual hallucinations or cognitive impairment, for those patients who exhibited them, and at the review of records for those without these symptoms. Similarly, age was defined as the age at symptoms onset for positive cases or the age at review of the records for negative cases.

Visual inspection of the parallel lines in the Log-Minus-Log survival plot (LML plot) was used to check the proportional hazards assumption for each covariate [31]. For this evaluation, quantitative covariates (i.e. age and years with L-DOPA) were categorized into tertiles.

The significance threshold was set to *p*<0.05. Odds (or Hazard) ratios and 95% confidence intervals were reported where relevant. All statistical analyses were performed using IBM SPSS 22 software for Windows.

## 3. Results

### 3.1. *GBA* mutation analysis in PD and HC

A total of 532 PD patients (55.4% male and 44.6% female; mean age 65 ± 11 years; mean age at onset 56 ± 12 years) and 542 HC (52.6% male and 47.4% female; mean age 62 ± 12 years) were successfully screened.

We detected a total of 43 variants across *GBA* sequences in PD and HC. In general, the frequency of *GBA* carriers was 12.2% for PD and 7.9% for HC (*p =* 0.021). None of the patients exhibited a homozygous state. However, a compound heterozygous state was found in 2.4% of PD patients and 1.6% of HC. The list of variants found, frequencies in PD and HC cohorts and its pathogenicity based on *in-silico* analysis is shown in detail in S1 Table. All variants were nucleotide substitutions, except one deletion. Nine variants were synonymous, 24 were non-synonymous, 10 were located in intronic areas and 1 was a frameshift deletion. The splicing process did not appear to be affected by intronic or exonic variants according to NSPLICE and HSF analyses. Of the 43 variants, 17 were previously reported (12 related to GD, 3 identified as PD risk factors, and 2 identified as both), 19 were novel and 7 were otherwise polymorphisms. The *in-silico* analysis revealed that 18 of these variants were potentially deleterious and the other 25 were potentially benign.

**Fig 1:**
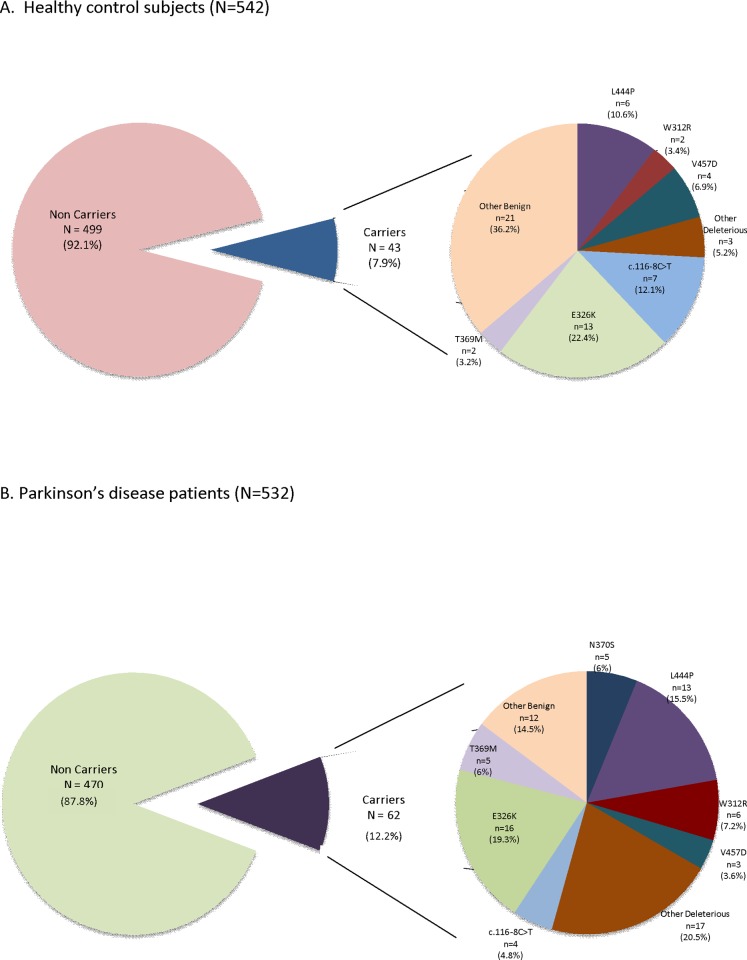
Pie plots showing the frequencies of the *GBA* variants found. A) control subjects and B) Parkinson’s disease patients. N = number of subjects. n = number of times in which a variant is present.

When classified by potential pathogenicity, the proportion of benign *GBA* variants was similar for PD and HC (5.3% vs. 5.5%; OR = 0.95; *p* = 0.87). Conversely, deleterious *GBA* variants were more prevalent in PD than in HC, and they correlated with an increased odds of PD (7.7% vs. 2.7%; OR = 2.96; *p*<0.001). Pie plots in Fig 1 show the frequencies of the *GBA* variants found separately for PD (A) and HC (B) groups.

### 3.2. Systemic features in PD and HC groups

In order to rule out Gaucher symptoms in those subjects with a compound heterozygous *GBA* state, we performed a detailed review of all the records belonging to these subjects. Only one PD patient exhibited chronic anaemia and splenomegaly besides a family history of dementia in both progenitors. The index of suspicion for GD was high; however, plasma chitotriosidase activity was 62.5nmol/ml/h and normal glucocerebrosidase activity was observed in dried blood spots 4.0μmol/L/h, and therefore the possibility of GD was dismissed. Within the HC group, one subject with a compound heterozygous *GBA* state had pancytopenia related to liver cirrhosis due to the hepatitis C viral infection and a family history of cirrhosis on his father’s side. The patient underwent a liver transplant and died at the age of 54. Unfortunately, data concerning plasma chitotriosidase and glucocerebrosidase activities was not available prior to the liver transplant, but there was no histological evidence of GD within the explanted liver. None of the remaining patients exhibited any other condition related to GD.

### 3.3. Comparison of clinical features in PD between Deleterious and Benign *GBA* carriers vs. non-carriers

#### 3.3.1. Univariate analyses

Descriptions and results for the comparisons of the clinical features across the three groups of PD patients (Deleterious *GBA*-carriers, Benign *GBA*-carriers and non-carriers) are included in Table 1. The mean disease duration at evaluation was >10 years for the three groups and there were no statistical differences. Age at disease onset was lower for Deleterious *GBA*-carriers than for non-carriers (50.6 vs. 56.6 years; *p* = 0.005). We found that tremors were less common in both Deleterious *GBA-*carriers (50% vs. 71%; OR = 0.41 (95% CI 0.21–0.81); *p* = 0.008) and Benign *GBA*-carriers (46% vs. 71%; OR = 0.35 (95% CI 0.15–0.8); *p* = 0.01). With regard to dopaminergic motor complications, we found that dyskinesias was more common in Deleterious *GBA*-carriers (61% vs. 42%; OR = 2.17 (95% CI 1.08–4.34); *p* = 0.026) and Benign *GBA*-carriers (78% vs. 42%; OR = 4.97 (95% CI 1.81–13.61); *p* = 0.001). Also, motor fluctuations and related phenomena (i.e. wearing-off, delayed-on; see Table 1) were more frequently observed in both Deleterious *GBA*-carriers (81% vs. 52%; OR = 3.95 (95% CI 1.7–9.71); *p* = 0.001) and Benign *GBA*-carriers (88% vs. 52%; OR = 6.45 (95% CI 1.9–21.9); *p* = 0.001). In terms of neuropsychiatric conditions, symptoms were more frequently observed only in Deleterious *GBA*-carriers. They demonstrated a higher prevalence, with respect to non-carriers, of cognitive impairment (40% vs. 18%; OR = 2.90 (95% CI 1.45–5.8); *p* = 0.002), visual hallucinations (47% vs. 22%; OR = 3.15 (95% CI 1.6–6.17); *p* = 0.001) and other similar conditions such as benign hallucinations, psychosis and temporal-spatial disorientation (Table 1).

**Table 1:** Demographic and clinical features of *GBA* variant carriers (Deleterious *GBA*-carriers and Benign *GBA*-carriers) and non-carriers.

		Deleterious GBA-carriers	Benign GBA-carriers	Non-carriers	P-value^1^
Demographics and onset					
	Sex (% men)	52.6	58.3	55.7	p = 0.89
	Age at onset (years± SD)	50.6± 9.4	53.6±10.6	56.6±12.9	**p = 0.005**/ p = 0.26
	Disease duration (years ± SD)	11.4±5.3	12.5±6.3	9.8±6.9	p = 0.16/ p = 0.07
	Main symptom at disease onset				p = 0.86
	•Tremor (%)	55.3	66.7	62	
	• Rigidity (%)	0	0	7	
	• Bradykinesia (%)	15.8	16.7	13.6	
	• Gait disorder (%)	5.3	0	6.4	
	• Pain (%)	5.3	4.2	4.2	
	• Change in writing (%)	0	4.2	2	
	• Other (%)	10.2	18.4	8.3	
Motor					
	Tremor (%)	50	45.8	70.7	**p = 0.008/ p = 0.01**
	Rigidity (%)	86.8	100	92.7	p = 0.19/ p = 0.17
	Bradykinesia (%)	94.6	91.7	95.5	p = 0.8/ p = 0.39
	Gait disorder (%)	82.9	83.3	76.9	p = 0.41/ p = 0.46
	Postural instability (%)	69.4	58.3	55.5	p = 0.1/ p = 0.78
	Falls (%)	31.4	33.3	26.1	p = 0.5/ p = 0.46
	Freezing (%)	38.2	36.4	37.3	p = 0.9/ p = 0.93
Dopaminergic complications					
	Dyskinesias (%)	61.1	78.3	42	**p = 0.026/ p = 0.001**
	Disease duration at onset of dyskinesias (years ± SD)	6.8±2.9	8.8±4.4	8.6±5.5	p = 0.14/ p = 0.88
	Years on L-DOPA at onset of dyskinesias (years ± SD)	3.7±2.5	6±2.2	5.2±4	p = 0.12/ p = 0.4
	Peak-dose dyskinesias (%)	61.1	73.9	40.6	**p = 0.016/ p = 0.002**
	Biphasic dyskinesias (%)	0	10.5	4.9	p = 0.17/ p = 0.27
	Motor fluctuations (%)	81.1	87.5	52	**p = 0.001/ p = 0.001**
	Disease duration at onset of motor fluctuations (years ± SD)	5.9±2.6	7.7±4.5	8.3±5.7	**p = 0.032**/ p = 0.59
	Years on L-DOPA treatment at onset of fluctuations (years ± SD)	2.7±1.7	5±3.2	4.4±3.6	**p = 0.025**/ p = 0.52
	Delayed-on (%)	64.9	75	43.4	**p = 0.001/ p = 0.002**
	Wearing-off (%)	75.7	83.3	48.1	**p = 0.012/ p = 0.001**
	No-on phenomenon (%)	22.9	22.7	15.2	p = 0.23/ p = 0.34
	Unpredictable off (%)	22.2	9.1	15.4	p = 0.28/ p = 0.42
	Dystonia (%)	34.2	26.1	25.1	p = 0.21/ p = 0.91
	Disease duration at onset of dystonia (years ± SD)	7.5±4.2	7.2±5.8	9.9±8.7	p = 0.36/ p = 0.55
	Years on L-DOPA at onset of dystonia (years ± SD)	4.3±3.5	7±6.4	4.7±4.8	p = 0.75/ p = 0.38
Neuropsychiatric					
	Cognitive impairment (%)	39.5	29.2	18.3	**p = 0.002**/ p = 0.19
	Disease duration at onset of cognitive impairment (years ± SD)	9.7±4.3	12±3.3	10.6±6.8	p = 0.64/ p = 0.59
	Mnestic failures without functional impairment (%)	36.8	45.8	30.8	p = 0.44/ p = 0.12
	Temporal-spatial disorientation (%)	25.7	8.3	11	**p = 0.01**/ p = 0.68
	Visual hallucinations (%)	47.4	29.2	22.2	**p = 0.001**/ p = 0.43
	Disease duration at onset of hallucinations (years ± SD)	9±5.2	10.8±6	9.3±6.7	p = 0.88/ p = 0.56
	Years with L-DOPA treatment at onset of hallucinations (years ± SD)	7±5.9	6.8±4.3	5.1±5.2	p = 0.23/ p = 0.44
	Benign hallucinations (%)	55.6	36.4	32.4	**p = 0.005**/ p = 0.7
	Auditory hallucinations (%)	2.9	0	2.1	p = 0.76/ p = 0.49
	Psychosis (%)	20	9.1	9.1	**p = 0.037**/ p = 1
	Impulse control disorders (%)	39.4	25	29.9	p = 0.27/ p = 0.65
	Obsessive-compulsive behavior (%)	11.8	0	15.7	p = 0.54/ **p = 0.049**
Mood					
	Depression (%)	41.7	47.8	38.1	p = 0.67/ p = 0.35
	Disease duration at onset of depression (years ± SD)	5.7±4.9	7.4±5.9	4.4±6.1	p = 0.54/ p = 0.15
	Anxiety (%)	29.4	34.8	29.8	p = 0.96/ p = 0.61
	Emotional lability (%)	3.1	5	12	p = 0.13/ p = 0.34
Autonomic					
	Orthostatic hypotension (%)	20	7.1	22.7	p = 0.75/ p = 0.17
	Constipation (%)	61.5	80	53.7	p = 0.44/ **p = 0.046**
	Urinary incontinence (%)	56.3	57.9	58	p = 0.85/ p = 0.9
	Sexual impotence (%)	16.7	12.5	20	p = 0.69/ p = 0.6
Other non-motor					
	REM sleep behaviour disorder (%)	69.4	70.8	50.1	**p = 0.026/ p = 0.049**
	Insomnia (%)	35.3	45.8	35.7	p = 0.96/ p = 0.31
	Hyposmia (%)	47.1	58.3	40	p = 0.59/ p = 0.23
	Dysphagia (%)	31	23.5	20.5	p = 0.18/ p = 0.76
	Dyspnoea (%)	0	0	6.7	p = 0.15/ p = 0.27
	Pain (%)	37.9	40	34.4	p = 0.7/ p = 0.61
	Sweating (%)	10.7	6.7	17.2	p = 0.38/ p = 0.29
Treatment					
	L-DOPA equivalent daily dose (dose ± SD) (mg/day)	922.5±471.4	1048.2±465.1	747.9±477.5	**p = 0.04/ p = 0.004**
	Response to L-DOPA (%)	93.3	100	98	p = 0.13/ p = 0.5

^1^ For the pair-wise comparisons, p-value is presented as deleterious vs non-carriers/benign vs non-carriers.

Other non-motor features of interest included a higher prevalence of REM sleep behaviour disorders (RBD) in both Deleterious *GBA*-carriers (69% vs. 50%; OR = 2.26 (95% CI 1.08–4.72); *p* = 0.026) and Benign *GBA*-carriers (71% vs. 50%; OR = 2.41 (95% CI 0.98–5.97); *p* = 0.049), and a higher prevalence of constipation in Benign *GBA*-carriers only (80% vs. 54%; OR = 3.45 (95% CI 0.95–12.46); *p* = 0.046). Lastly, regarding treatment, we found that Deleterious *GBA*-carriers and Benign *GBA*-carriers require a higher daily equivalent dose of L-DOPA (923 and 1048 vs.748 mg/day; *p* = 0.04).

#### 3.3.2. Multivariate analyses

**Fig 2:**
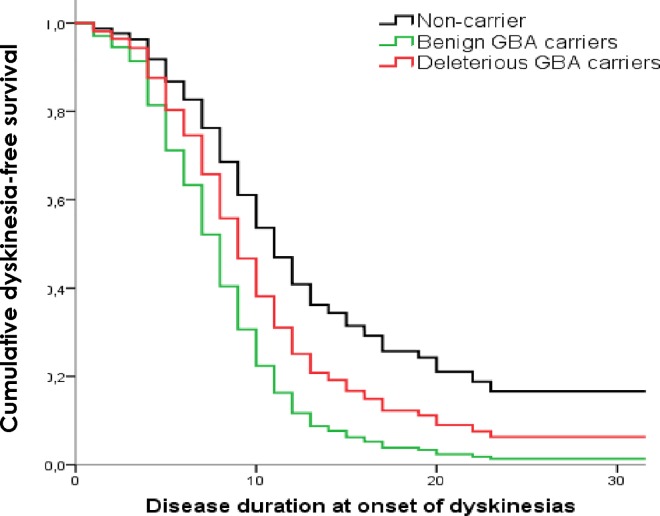
Kaplan-Meier plot of dyskinesias onset. Lines represent the cumulative dyskinesias-free survival in years from disease onset.

**Fig 3:**
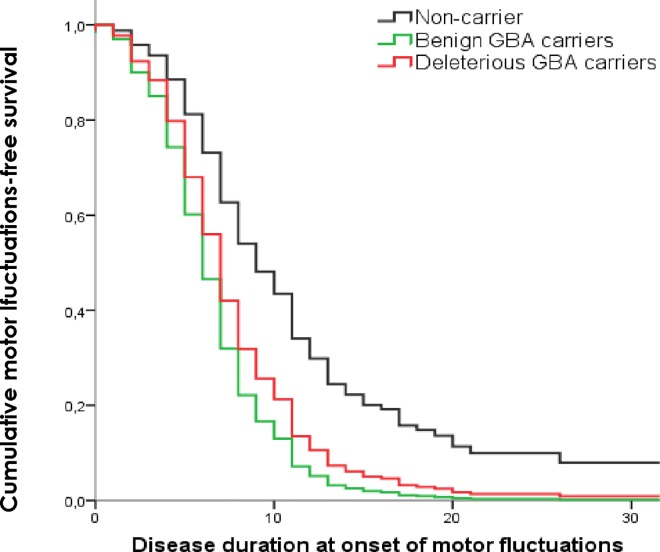
Kaplan-Meier plot of motor fluctuations onset. Lines represent the cumulative motor fluctuations-free survival in years from disease onset.

**Fig 4:**
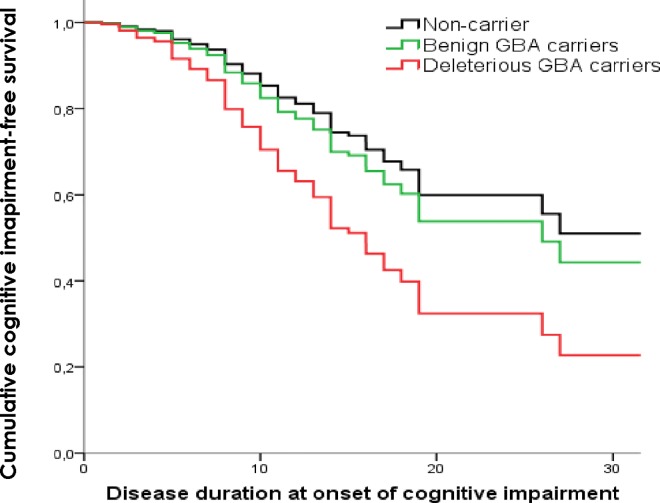
Kaplan-Meier plot of cognitive impairment onset. Lines represent the cumulative cognitive impairment-free survival in years from disease onset.

**Fig 5:**
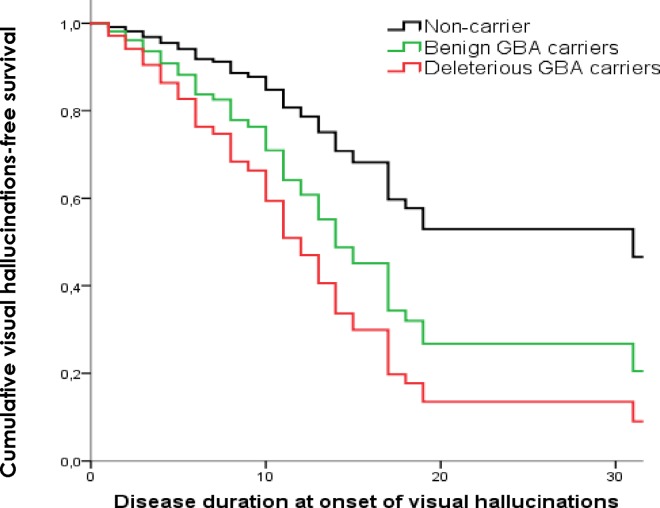
Kaplan-Meier plot of hallucinations onset. Lines represent the cumulative hallucinations-free survival in years from disease onset.

Data included in the multivariate analysis was collected systematically. Information concerning the presence of dyskinesias was collected in 99.2% of PD cases, motor fluctuations collected in 98.9% and cognitive impairment in 95.7%. The survival analysis results are presented in S2 Table. Accounting for *GBA* status, Cox regression mainly revealed that:

1. the development of dyskinesias is influenced over time by the presence of benign *GBA* variants (HR = 2.4 (95% CI 0.95–2.51); *p* = 0.001) (Fig 2),
2. the presence of motor fluctuations is influenced by the presence of deleterious (HR = 1.85 (95% CI 1.22–2.81); *p* = 0.004) and benign *GBA* variants (HR = 2.44 (95% CI 1.51–3.96); *p*<0.001) (Fig 3),
3. the progression of the disease to cognitive impairment is influenced by the presence of deleterious *GBA* variants (HR = 2.6 (95% CI 1.25–3.88); *p* = 0.001) (Fig 4) and
4. the development of visual hallucinations is influenced by the presence of deleterious *GBA* variants (HR = 3.15 (95% CI 1.71–5.79); *p*<0.001) (Fig 5).

## 4. Discussion

In this study we have performed a complete mutational screening of *GBA* in a large cohort of PD patients and controls from southern Spain. PD patients had a higher frequency of deleterious variants than controls, but a similar proportion of benign variants. As in previous studies, PD patients carrying Deleterious *GBA* were younger at disease onset and more likely to suffer from neuropsychiatric disturbances [9–11]. As a new finding, we have observed that PD patients carrying both Deleterious and Benign *GBA* have higher rates of progression to treatment-related motor complications.

The frequency of *GBA* variants in PD in our work was 12.2%, slightly higher than the 9.8% found in 225 Spanish PD patients from another study [11]. Previous studies performing full *GBA* sequencing in European populations reported lower frequencies: 8.3% in 230 Portuguese subjects [2], 6.4% in 172 Greek subjects [32], 4.2% in 790 British subjects [6], and 6.9% in the multicenter analysis of 4911 non-Ashkenazi PD patients [7]. The same applies for HC, for whom the frequency is usually ~1%. However, in order to compare the frequencies fairly, one must take into account that our frequencies refer to all types of *GBA* variants, and inclusion criteria for a variant in this calculation can differ across studies. Indeed, in this study, if only deleterious variants are considered, the frequencies drop to 7.9% and 2.7% for PD and HC respectively. In any case, it seems that the prevalence of *GBA* variants in Spain is slightly higher than the average for other European countries, possibly due to a diversity in the genetic background in the Iberian Peninsula [33].

In the last years, several subtypes of PD have been described based on the clinical phenotype of patients [34]. In this study, we observed that both groups of *GBA* variant-carriers (benign and deleterious variants) had less tremor, suggesting that these carriers might belong to the non tremor-dominant phenotype of PD [34]. This suggestion could be in accordance to the fact that non-tremor dominant phenotype group tend to present a higher risk of developing a cognitive decline as our patients with *GBA* deleterious variants [35]. However, it should be verified through prospective studies.

*GBA* variant-carriers also had a higher prevalence of treatment-related motor complications, such as dyskinesias and motor fluctuations and they tended to develop them earlier than non-carriers. This result indicates that *GBA* status might well be a prominent risk factor influencing the development of treatment-related motor complications. This association was still significant after correcting for other risk factors such as prolonged treatment with L-DOPA, dose and age at disease onset. This is the first time, to our knowledge, that such a wide variety of dopamine-related features has been related to *GBA*. This observation contrasts to recent findings obtained by Oeda et al [12]. On their work, *GBA* status, either GD mutations and GD non-related mutations, were not linked to treatment-related motor complications. Nevertheless, our study accounts for a larger sample size and our results could therefore establish more reliable data compared to the aforementioned study. In additon, the motor complications usually appear conjointly, as in our study, since they are a result of the pulsatile stimulation of dopamine receptors by the dopaminergic replacement therapy [36].

On the other hand, we are not the first to suggest that non-pathogenic variants in *GBA* could affect motor symptomatology. Winder-Rhodes et al. observed that not only pathogenic mutations in *GBA* but also polymorphisms carried an increased risk of progression to H&Y Stage 3 in PD patients [14]. Although their results must be verified using larger samples, our results reinforce the hypothesis that (theoretically) potentially benign variants in *GBA* play a role in motor disease progression since motor complications linked to dopamine replacement therapy can be influenced by their presence. If so, *GBA* status should be an important factor to account for in the therapy of PD patients. Further research on this finding is therefore needed in order to address this issue.

Earlier disease onset and a higher prevalence of cognitive impairment or dementia and visual hallucinations in PD patients with *GBA* mutations have been reported on extensively in scientific literature [9–11]. Brockmann et al. longitudinally observed a more rapid progression in cognitive decline in 20 carriers of L444P or N370S mutations [13]. Similarly, Setó-Salvia et al. observed an increased risk of dementia in a group of 22 PD patients, of which the majority carried pathogenic *GBA* mutations [11]. In our study we reaffirmed these observations but, interestingly, only in patients with deleterious mutations. Only PD patients with potentially deleterious variants had a lower age of disease onset, and in the survival analysis we observed that the development of cognitive impairment was accelerated by the presence of deleterious mutations, and that an older age of disease onset was also a risk factor. In accordance with our results, Winder-Rhodes et al. observed that progression to dementia was accelerated for pathogenic *GBA* mutation-carriers but not for *GBA* polymorphism-carriers [14]. Moreover, we have observed this discrepancy between Benign and Deleterious *GBA*-carriers in progression to visual hallucinations. Not surprisingly, older age at disease onset and a prolonged L-DOPA treatment, factors that are known to be influential, were also independent risk factors for this accelerated development. [29]. Other similar findings included an increased level of psychosis, benign hallucinations and temporal-spatial disorientation. Dementia and visual hallucinations are conditions that typically occur in advanced stages, and are possibly related to the presence of Lewy bodies in cortical areas. In fact, autopsies of *GBA* mutation-carriers revealed a more widespread Lewy body pathology in the neocortex than that of non-carriers [6] and an increased rate of *GBA* mutations in Parkinson’s disease with dementia and Lewy body disease [37]. On the basis of our results we therefore hypothesize that deleterious *GBA* variants may lead to a major alteration to the molecular mechanisms involved in α-synuclein clearance. Consequently, α-synuclein brain pathology could reach cortical areas faster. Benign variants might also play a role, although their functionality and severity may differ.

Patients with deleterious and benign variants were also more likely to suffer from RBD. Conversely, a previous study found no statistical difference in sleep quality measured by Parkinson Disease Sleep Scale (PDSS) between N370S or L444P mutation carriers and non-carriers [9]. Nonetheless, PDSS quantifies various aspects of sleep disturbance beyond REM sleep and therefore our results are not directly comparable. A better study design including diagnosis of RBD through polysomnography would therefore be advisable to reaffirm this finding. Lastly, patients with benign variations more frequently suffered from constipation. Some autonomic feature dysfunctions have been observed in a group of N370S or L444P mutation carriers [9]. These authors did not investigate constipation, but reported an association between *GBA* status and orthostatic, urinary and sexual functions. We have not reproduced these particular findings; however 80% of our patients suffered constipation. Autonomic symptom dysfunction therefore could be over-represented in these carriers

Our study also has potential limitations. The possible pathogenicity of the found variants was based on bioinformatic analysis. In accordance with this method, we classified E326K and T369M as potential benign variants. The role of E326K has been discussed in recent years. There is some evidence to suggest that E326K in a compound heterozygous state can act as a modifying factor, eventually inducing a pathogenic effect. Nevertheless, its own role in homozygous and single heterozygous states remains controversial [38]. A recent work developed by Alcalay et al. shows a lower glucocerebrosidase enzymatic activity in those subjects carrying E326K than in the group of non-mutation carriers [39]. However, this comparison included a heterogeneous group, gathering as carriers PD patients and control participants, despite having previously found statistical differences between them. In addition, the reduction in glucocerebrosidase activity associated with E326K was lower than that linked to known pathogenic variants such as L444P, suggesting that it alters the protein function to a lower degree. On the other hand, the impact of E326K on the cognitive status in PD has been recently assessed [40]. It seems that presence of E326K might influence in some cognitive domains performance. However, E326K carriers had the lowest risk of cognitive impairment compared to GD causing mutations carriers. Due to these facts, bioinformatic tools might categorize this slight difference associated with E326K as non-deleterious and it can explain the classification of E236K as a benign variant following our methodology. Besides, in our study we found a similar frequency of E326K in PD and HC, providing evidence for its possibly benign role. On the basis of such observations, we think that the role of this variant should be still objective of further investigations to settle robust conclusions and further functional studies should be desirable.

On the other hand, the genetic assessment has some limitations. HRM fails to accurately identify copy number variations and homozygous state. However, neither deletions, nor insertions in *GBA* have been linked to PD and combination of DNA sequencing was done to overcome the identification of homozygous cases.

We observed that the phenotypic assessment had some missing data. Nevertheless, apart from hyposmia, the mean of collected data from the phenotypic evaluation reached 88%. This high percentage of available information from a sample as large as ours could partially compensate for this deficiency and allow for reliable conclusions. On the other hand, although levodopa requirements for the onset of visual hallucinations and motor complications were not collected, we included additional information. Since years on L-DOPA and disease duration are influential factors in the genesis of this symptomatology, they were controlled. However, for future studies missing data regarding L-DOPA dose should be recorded in order to facilitate an optimal assessment and to clarify their influence in the development of these features.

Finally, we acknowledge that a prospective longitudinal design, using validated scales to evaluate motor and non-motor symptoms, is better in assessing disease progression. Conversely, a potential strength is our large sample of *GBA* variant carriers, which improves the reliability of our results. We expect to make the most of this favorable sample size in the future by working further on the hypotheses that have emerged from this study.

In conclusion, in this study we have observed that *GBA* variants have a different impact on the PD phenotype according to its pathogenicity. Motor symptoms and treatment-related motor complications could be most likely influenced by all types of *GBA* variants, whereas the expression of non-motor symptoms could be instead related to the presence of deleterious variants. In view of these results, we would like to stress the importance of fully screening *GBA* since up to 75% of the variants in this PD sample were not L444P and N370S mutations, and they had an effect on the phenotype. These findings have prognostic value for the clinician and improve understandings of the functionality of *GBA*.

## Supporting Information

S1 Table List of *GBA* variants found in a southern Spanish population and *in-silico* assessment of its pathogenicity by bioinformatic tools. *Number of times in which a variation is present in each group.
(DOC)

S2 Table Cox regression analysis. Results of Cox regression analysis of *GBA* variant status, sex, age and years with L-DOPA (except for cognitive impairment and depression) as predictor variables for progression to motor fluctuations, dyskinesias, cognitive impairment, visual hallucinations and depression.
(DOCX)
