# Supplementary material for: GBA Variants Influence Motor and Non-Motor Features of Parkinson’s Disease
Source: PLoS One. 2016 Dec 28;11(12):e0167749. doi: 10.1371/journal.pone.0167749 (PMC5193380; doi:10.1371/journal.pone.0167749)
Supplement: S1 Table — *Number of times in which a variation is present in each group. (DOC) [file pone.0167749.s001.doc]

**S1 Table 1. List of *GBA* variants found in a southern Spanish population and *in-silico* assessment of its pathogenicity by bioinformatic tools.**

*Number of times in which a variation is present in each group

Potential deleterious Potential benign

NA: not available PD: Parkinson's disease GD: Gaucher disease HC: Healthy control PSA: Potential splicing acceptor
