# Supplementary material for: GBA Variants Influence Motor and Non-Motor Features of Parkinson’s Disease
Source: PLoS One. 2016 Dec 28;11(12):e0167749. doi: 10.1371/journal.pone.0167749 (PMC5193380; doi:10.1371/journal.pone.0167749)
Supplement: S2 Table — Results of Cox regression analysis of GBA variant status, sex, age and years with L-DOPA (except for cognitive impairment and depression) as predictor variables for progression to motor fluctuations, dyskinesias, cognitive impairment, visual hallucinations and depression. (DOCX) [file pone.0167749.s002.docx]

**S2 Table 1. Cox regression analysis.**

|  |  | P-value | Hazard Ratio (95% CI) |
| --- | --- | --- | --- |
| Dyskinesias |  |  |  |
|  | Deleterious *GBA*-carriers vs. non-carriers | 0.078 | 1.54 (0.95-2.51) |
|  | Benign *GBA*-carriers vs. non-carriers | 0.001 | 2.4 (1.41-4.09) |
|  | Sex | <0.001 | 0.59 (0.44-0.79) |
|  | Age | <0.001 | 0.97 (0.96-0.98) |
|  | Years with L-DOPA | 0.061 | 0.97 (0.94-1) |
| Motor fluctuations |  |  |  |
|  | Deleterious *GBA*-carriers vs. non-carriers | 0.004 | 1.85 (1.22-2.81) |
|  | Benign *GBA*-carriers vs. non-carriers | <0.001 | 2.44 (1.51-3.96) |
|  | Sex | 0.16 | 0.73 (0.56-0.94) |
|  | Age | <0.001 | 0.97 (0.96-0.98) |
|  | Years with L-DOPA | 0.017 | 0.96 (0.93-.099) |
| Cognitive impairment |  |  |  |
|  | Deleterious *GBA*-carriers vs. non-carriers | 0.006 | 2.2 (1.25-3.88) |
|  | Benign *GBA*-carriers vs. non-carriers | 0.63 | 1.21 (0.55-2.63) |
|  | Sex | 0.69 | 0.92 (0.61-1.38) |
|  | Age | 0.002 | 1.03 (1.01-1.05) |
| Visual hallucinations |  |  |  |
|  | Deleterious *GBA*-carriers vs. non-carriers | <0.001 | 3.15 (1.71-5.79) |
|  | Benign *GBA*-carriers vs. non-carriers | 0.09 | 2.07 (0.87-4.91) |
|  | Sex | 0.032 | 0.62 (0.4-0.96) |
|  | Age | 0.003 | 1.03 (1.01-1.05) |
|  | Years with L-DOPA | <0.001 | 0.90 (0.85-0.93) |
| Depression |  |  |  |
|  | Deleterious *GBA*-carriers vs. non-carriers | 0.773 | 0.90 (0.46-1.79) |
|  | Benign *GBA*-carriers vs. non-carriers | 0.25 | 1.46 (0.76-2.8) |
|  | Sex | 0.11 | 0.75 (0.52-1.7) |
|  | Age | <0.001 | 0.972 (0.96-0.98) |

Results of Cox regression analysis of *GBA* variant status, sex, age and years with L-DOPA (except for cognitive impairment and depression) as predictor variables for progression to motor fluctuations, dyskinesias, cognitive impairment, visual hallucinations and depression.
